# Sintilimab-induced myocarditis suspected in a patient with esophageal cancer and followed septic shock: case report and literature review

**DOI:** 10.3389/fonc.2024.1465395

**Published:** 2024-09-16

**Authors:** Qihao Zhou, Zhiquan Qin, Guoqing Wu, Peiyuan Yan, Qunjiang Wang, Jing Qu, Jiahong Jiang, Da Ye

**Affiliations:** Cancer Center, Department of Medical Oncology, Zhejiang Provincial People’s Hospital, Affiliated People’s Hospital, Hangzhou Medical College, Hangzhou, Zhejiang, China

**Keywords:** case report, sintilimab, myocarditis, immune-related adverse events, methylprednisolone, septic shock

## Abstract

**Background:**

Immune checkpoint inhibitors (ICIs) have become a prevalent tool in anti-tumor therapy in recent years. They may cause immune-related adverse events (irAEs) including potentially life-threatening cardiovascular toxicities such as myocarditis.

**Case presentation:**

In this report, we describe a 69-year-old man with recurrent esophageal cancer who developed myocarditis after receiving three cycles of sintilimab combined with nab-paclitaxel. Despite a rising cardiac troponin I (cTnI), he initially reported no discomfort. He was later suspected of having with sintilimab-induced myocarditis. Although treatment with methylprednisolone reduced his cTnI levels, he still experienced significant discomfort. Moreover, he developed pneumonia and septic shock.

**Conclusion:**

In our literature search to identify all reported cases of sintilimab-associated adverse events involving myocarditis, we found 14 patients, including those with esophageal cancer, thymoma, lung cancer, gastric cancer, hepatobiliary carcinoma, and chordoma. The primary treatment for ICI-induced cardiotoxicity is methylprednisolone. However, the long-term or high-dose use of steroids can also induce side effects, which have not been the focus of these case reports. This is the first reported case of asymptomatic immune-mediated myocarditis occurring during the treatment of esophageal cancer with sintilimab. It is also the first to address the side effects of methylprednisolone used in the treatment of sintilimab-related myocarditis. To facilitate an early diagnosis, regular monitoring is required during sintilimab treatment. We should also focus on the prevention and management of adverse effects related to steroid use.

## 1 Introduction

Immune checkpoint inhibitors (ICIs) have emerged as promising anti-tumor therapies in recent years. However, they can cause distinct immune-related adverse events (irAEs), owing to the inhibition of immunological inhibitory mechanisms. Cardiovascular irAEs are more common than were initially reported in early ICI clinical trials (1). ICIs induced cardiovascular toxicities, including myocarditis, pericarditis, arrhythmias, cardiomyopathy, and acute coronary syndrome. Of these, myocarditis has been the most extensively studied due to its incidence and severity. The mortality attributed to ICI-myocarditis ranges from 36% to 67% (2–4). Sintilimab, a programmed cell death-1 (PD-1) inhibitor, has been approved for various cancers in China, including esophageal cancer. We searched for reports on sintilimab-related adverse events involving myocarditis (5–18), identifying 14 patients. The primary strategy for managing ICI-induced cardiotoxicity is immunosuppressive therapy (6). However, long-term or high doses of steroid use can also induce side effects, which were outside the scope of these case reports. Herein, we reported a 69-year-old man with recurrent esophageal squamous cell carcinoma, who received sintilimab combined with nab-paclitaxel for 3 cycles. His cardiac troponin I (cTnI) continued to increase significantly; however he had not complained of any discomfort. After treatment with methylprednisolone, his cTnI levels decreased, but he experienced multiple discomforts, such as leg cramps, weakness while walking, sore throat, elevated blood glucose, and even he developed a septic shock. This is the first reported case of asymptomatic immune-mediated myocarditis occurring during the treatment of esophageal cancer with sintilimab. It is also the first report to address the side effects caused by methylprednisolone in the treatment of sintilimab-related myocarditis. During the sintilimab treatment phase, regular monitoring is essential for early diagnosis. Additionally, managing and preventing the side effects associated with steroid should use should also be our primary focuses.

## 2 Case presentation

**Figure 1:**
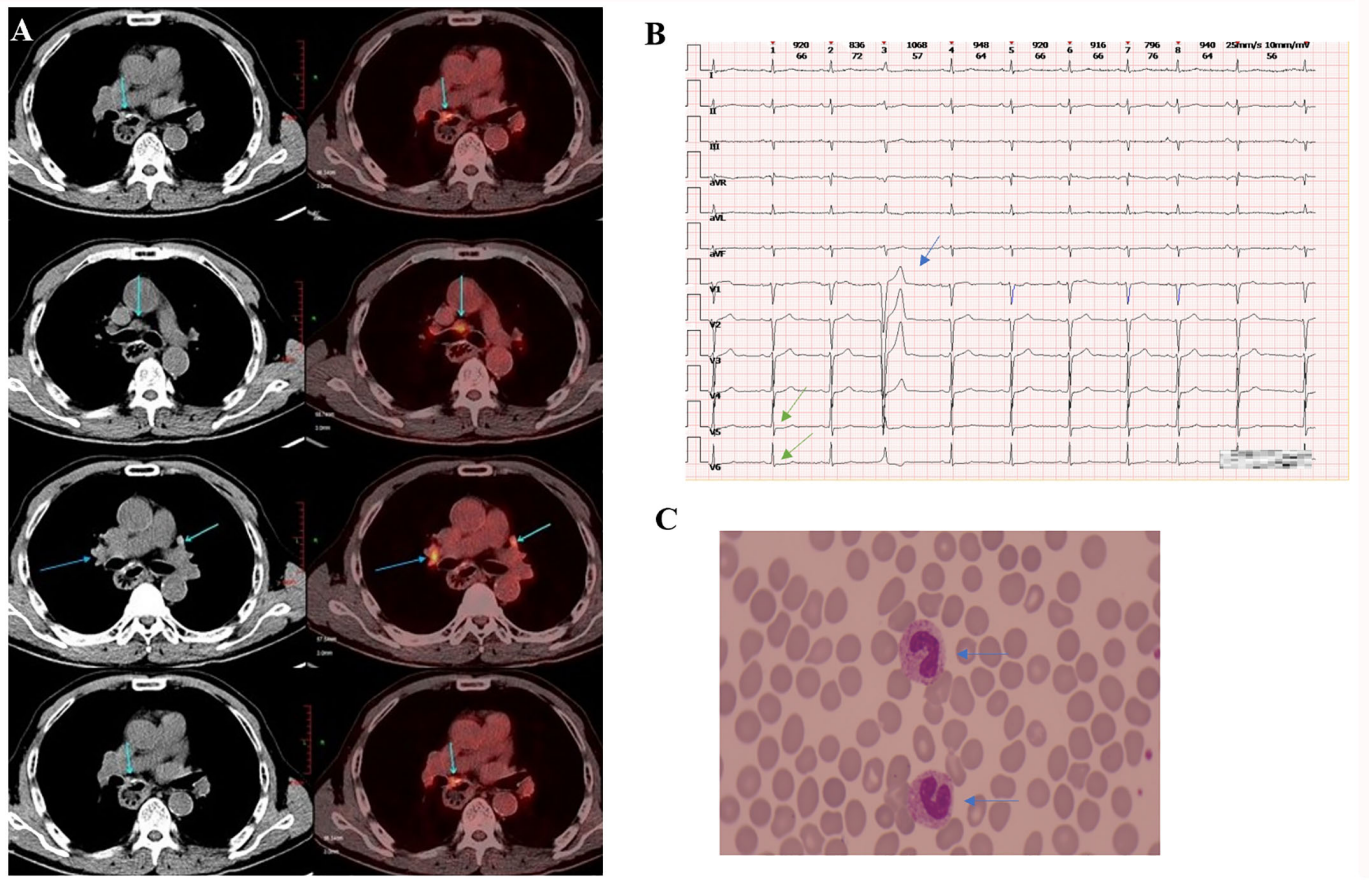
Clinical data of patients. **(A)** The PET/CT scan (taken in late February 2023) showed a soft tissue mass adjacent to the left side of the esophageal hiatus and above the retroperitoneal abdominal trunk, exhibiting increased FDG metabolism. **(B)** The ECG (taken in early June 2023) revealed mild ST segment depression in leads V5 and V6 (marked by the green arrow) and T-wave changes (indicated by the blue arrow), premature atrial contractions, short strands of atrial tachycardia, and premature ventricular contractions. **(C)** The blood smear examination (taken in late June, 2023) indicates a left shift in neutrophil nuclei.

A 69-year-old man with hypertension and diabetes presented at our hospital in June 2021, reporting dysphagia that had started two months earlier. External pathological examination revealed esophageal squamous cell carcinoma (ESCC). He underwent surgery in mid-June 2021, which confirmed the diagnosis of ESCC, staged at T3N1M0. He received four cycles chemotherapy with nab-paclitaxel (CSPC, OUYI, Pharmaceutical Co, Ltd) and nidaptin (Nanjing Xiansheng Dongyuan Pharmaceutical Co., Ltd) from August to October 2021. In February 2023, during a follow-up visit to the hospital, a positron emission tomography/computed tomography (PET/CT) scan showed a soft tissue mass adjacent to the left side of the esophageal hiatus and above the retroperitoneal abdominal trunk, exhibiting increased fluorodeoxyglucose (FDG) metabolism. This indicated a potential tumor recurrence or metastasis, possibly affecting the abdominal trunk (**Figure 1A**). From March to April 2023, he received three cycles of nab-paclitaxel (300 mg at Day 1), and sintilimab (Innovent Biologics Inc.) (200mg at Day 1).

When the patient was scheduled for his fourth cycle of treatment in late May 2023, the baseline cTnI monitoring revealed an abnormal elevation (cTnI 1.85μg/L, normal ≤0.050μg/L). Other cardiac markers, including creatine kinase (CK), B-type natriuretic peptide (BNP), lactate dehydrogenase (LDH), and D-dimer were normal. The electrocardiogram (ECG) indicated a sinus rhythm with a left deviation of electrical axis -35° and low QRS complex voltage in limb leads. An echocardiography showed mild aortic regurgitation, moderate mitral and tricuspid regurgitation, with no abnormalities in left ventricular diastolic and systolic function. The following day his cTnI significantly increased to 4.145μg/L. The patient had not complained of any discomfort. He was administered glucocorticoids (methylprednisolone 40 mg/day intravenous) for 3 days. However, his cTnI level further increased to 6.384μg/L. He declined the cardiac magnetic resonance (CMR) as he did not experience any heart-related symptoms. The dose of methylprednisolone was increased to 120 mg/day and administered for 3 days. Subsequently, the cTnl level decreased to 3.013μg/L but later increased to 4.904μg/L. A repeat ECG revealed mild ST-segment depression and T wave changes, premature atrial contractions, short strands of atrial tachycardia, and premature ventricular contractions (**Figure 1B**). An echocardiography indicated an ejection fraction (EF) of 60% and left atrial enlargement. Concurrently, the patient began experiencing chest tightness.

**Figure 2:**
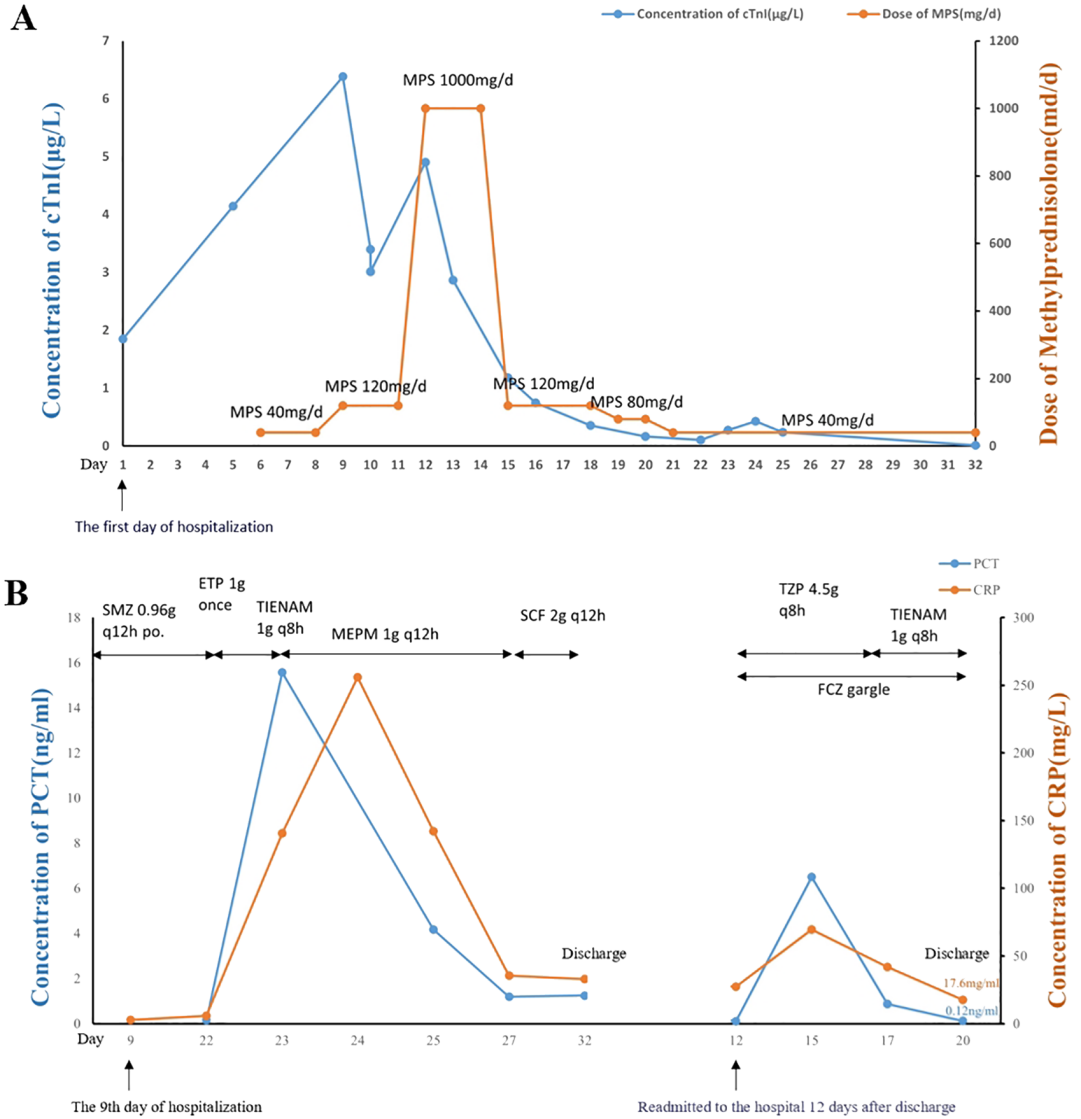
Changes in biochemical markers**. (A)** Levels of Troponin I before and after methylprednisolone treatment. When the MPS dose was adjusted to 120mg/d, cTnI showed a brief decrease, but then increased again. When the MPS dose was 1000mg/d, it was found that cTnI significantly decreased, and as the MPS dose decreased, cTnI did not rebound and gradually returned to normal levels. **(B)** Levels of PCT and CRP before and after antibiotic therapy. With the adjustment of antibiotics, the inflammatory markers CRP and PCT of the patient gradually decreased. However, discontinuation of medication during discharge led to a further increase in inflammatory markers, which were reduced when antibiotics were reused. cTnI, cardiac troponin I; MPS, methylprednisolone; d, day. PCT, procalcitonin; CRP, C-reactive protein; po, per os; q8h, every 8 hours; q12h, every 12 hours; SMZ, compound sulfamethoxazole tablets; ETP, ertapenem; TIENAM, imipenem and cilastatin sodium; MEPM, meropenem; SCF, sulbactam and cefoperazone; TZP, piperacillin sodium and tazobactam sodium; FCZ, fluconazole.

After ruling out immune pneumonia and acute coronary events, and considering the correlation between the sintilimab use and the examination results, a cardiology consultation strongly suspected sintilimab-induced myocarditis. High-dose steroids, specifically methylprednisolone pulse dosing 1000 mg/day, was administered intravenously for 3 days. The dose was then tapered to 120 mg/day for 4 days, 80mg/day for 2 days, and finally 40mg/day. To prevent infection, compound sulfamethoxazole tablets (SMZ) 0.96g were given orally every 12 hours (q12h). His cTnI decreased to 0.169 μg/ L (**Figure 2A**). During this period, he experienced cramps in his lower limbs, unsteady walking, sore throat and hyperglycemia.

**Figure 3:**
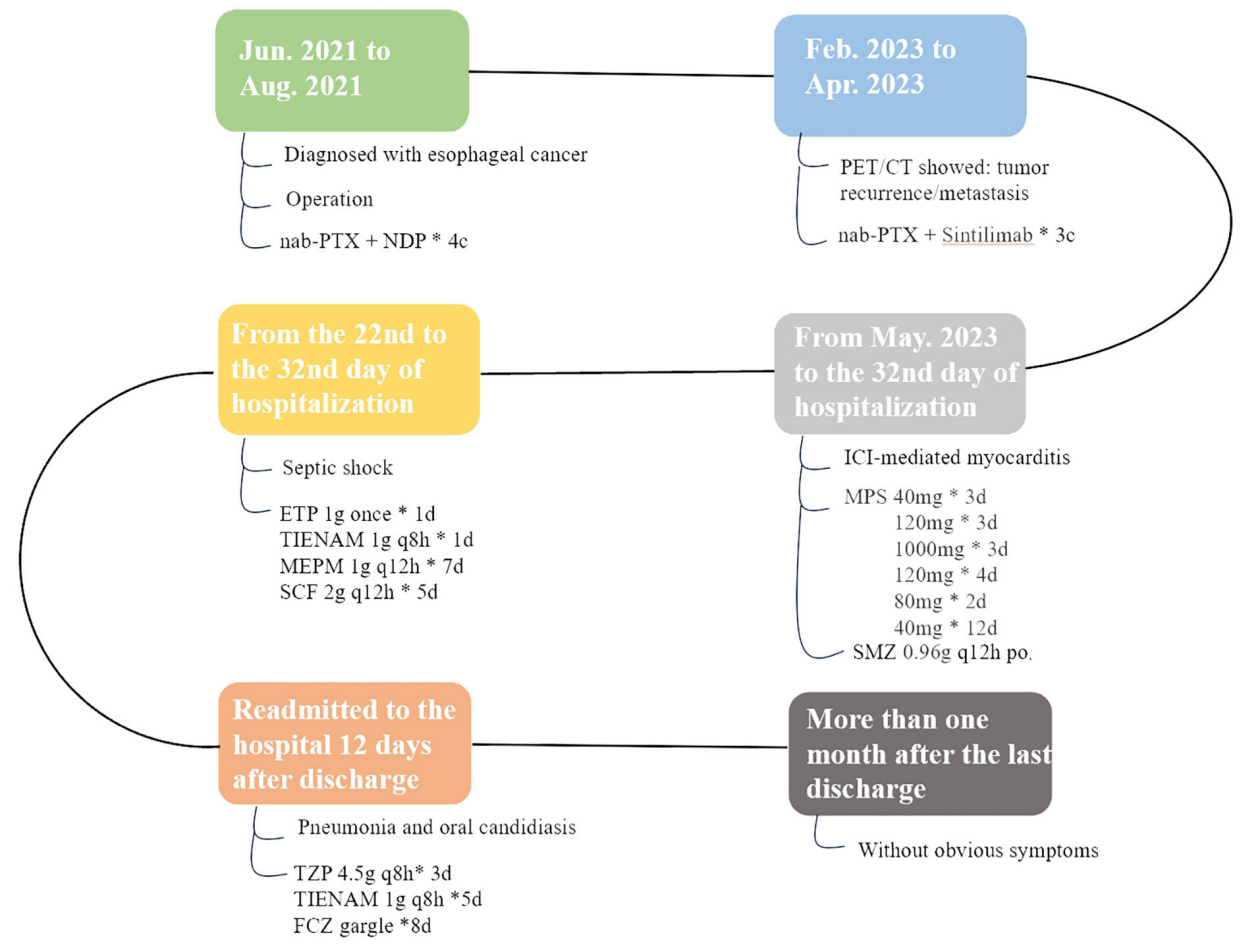
The treatment chart of the patient. nab-PTX, nab-paclitaxel; NDP, Nedaplatin; c, cycle; PET/CT, positron emission tomography/computed tomography; ICI, immune checkpoint inhibitors; MPS, methylprednisolone; d, day; po, per os; q8h,every 8 hours;q12h,every 12 hours;SMZ, compound sulfamethoxazole tablets; ETP, ertapenem; TIENAM, imipenem and cilastatin sodium; MEPM, meropenem; SCF, sulbactam and cefoperazone;TZP, piperacillin sodium and tazobactam sodium; FCZ, fluconazole.

On Day 16 of methylprednisolone treatment (the 22nd day of hospitalization), the patient developed a fever of 39.9°C with chills, and was subsequently treated with ertapenem 1g. The next day, he had persistent fever (38.1°C) and tachypnea. Labs showed leukocytosis (white blood cells of 25.01x10^9/L), neutrophilia (24.73x10^9/L), elevated C-reactive protein (CRP) (140mg/L), increased procalcitonin (PCT) (15.57ng/ml), reduced PaO_2_/FiO_2_ (163.5 mmHg), and high lactic acid (2.8 mmol/L). CT scan indicated scattered lung inflammation. We initiated treatment with imipenem and cilastatin sodium (TIENAM) at 1g every 8 hours (q8h). Subsequently, the patient experienced fatigue and his blood pressure dropped to 80/40mmhg. Considering these symptoms indicative of septic shock, we immediately started pressor support and fluid resuscitation. He was then transferred to the infection department for continued treatment. The blood smear indicated infection, evidenced by left shift of nucleus (**Figure 1C**). The patient was treated with meropenem 1 g q12h for 7 days, and with sulbactam and cefoperazone (SCF) 2 g q12h for 5 days. His PCT level decreased to 0.12ng/ml, and his CRP decreased to 17.6mg/L (**Figure 2B**). The patient’s symptoms improved, and vital signs were relatively stable. He was discharged after 32 days of hospitalization. However, the patient was readmitted to the hospital 12 days after his discharge due to oral pain and fever, where he was diagnosed with pneumonia and oral candidiasis. Fluconazole was administered for gargling. He received piperacillin sodium and tazobactam sodium (TZP) 4.5g q8h for 3 days, followed by TIENAM 1g q8h for 5 days. He was discharged after 8 days of admission. After more than one month of follow-up, the patient reported no significant discomfort. **Figure 3** provides a summary the treatment for this patient.

From the patient’s perspective, the adverse events (AEs) associated with ICI did not cause significant discomfort, unlike the side effects of methylprednisolone, which notably distressed him. Prompt anti-infection measures, along with attentive care from the medical staff, helped establish a rapport with him, contributing significantly to alleviating his anxiety.

## 3 Discussion and literature review

In recent years, ICIs for cancer have emerged as a potentially powerful therapeutic approach. Sintilimab is a recombinant human immunoglobulin G4 monoclonal antibody that targets PD-1. According to the ORIENT-15 trial (19), sintilimab when used in combination with chemotherapy, demonstrated significant advantages in both overall survival and progression-free survival for patients with ESCC. ICIs, however, carry the risk of disrupting immunological balance, leading to the development of autoreactive T cells and other irAEs. In the report (19), the most frequent adverse events related to treatment and classified as grade 3 or worse included a decrease in neutrophil count, decrease in white blood cell count, anaemia, and hypokalemia. Notably, immune myocarditis occurred in 1 case (0.3%) of the placebo-chemotherapy group, not in sintilimab-chemotherapy group (19). Although cardiovascular toxicity is a rare irAE, it has been reported frequently with the widespread use of ICIs. ICIs induce cardiovascular toxicities, including myocarditis, pericarditis, arrhythmias, cardiomyopathy, and acute coronary syndrome. Because of its onset and severity, myocarditis has been the most extensively investigated. The mortality rate attributed to ICIs-myocarditis is high, ranging from 36% to 67% (2–4).

The pathogenesis of ICI-associated myocarditis is currently unknown. Won et al. generated a clinically relevant mouse model for ICI-associated myocarditis by administering an αPD-1 monoclonal antibody (mAb). They found that the number and activity of cardiac-myosin-specific T cells were increased in these mice, indicating a contribution of heart autoantigen-reactive T cells to ICI-associated myocarditis. PD-1-expressing cardiac-myosin-specific T cells are present in the heart during naive conditions, and these T cells are under control by the PD-1/PD-L1 pathway in naive conditions, leading to the development of myocarditis (20). The study conducted by Matsumori revealed that the disruption of the PD-1 receptor, an inhibitory receptor found on T cells, resulted in fatal myocarditis in mice (21).

We conducted a comprehensive search of PubMed to identify all reported cases of sintilimab-associated myocarditis, as detailed in references (5–18). After reviewing all cases and excluding duplicate reports, the clinical features, treatment, and outcomes of these patients were compiled in **Table 1**. Among the 14 patients, 3 had thymoma. 6 had non-small cell lung cancer, 1 had gastric cancer.1 had extrahepatic cholangiocarcinoma, 1 had hepatocellular carcinoma, 1 had chordoma and 1 had esophageal cancer. Despite the report by Hong et al. (18), a patient with esophageal cancer treated with sintilimab developed inflammatory myopathy, which also exhibited signs of myocarditis. The immune inflammation reported in their case involves multiple areas. Our patient, however, had asymptomatic myocarditis, and although the course of the disease was potentially dangerous, its onset was insidious. Among the reported cases, some primarily presented with extensive myositis (8, 10, 17, 18). Additionally, several cases were associated with myasthenia gravis (6, 8, 10, 15). Furthermore, one case was complicated by autoimmune hepatitis (11), while another had rhabdomyolysis (17).Immune myocarditis can occur at any time during sintilimab therapy, with the longest interval (5) being 4 months after 5 courses, and the shortest (10) being 1 week after 1 course. However, most cases developed myocarditis after the first course of sintilimab administered (6–8, 10–15). In our case, immune myocarditis occurred 3 weeks after 3 cycles.

**Table 1:** Characteristics of the reported cases of sintilimab-induced myocarditis.

Author,year	Age/sex	PT	Onset of myocarditis	Clinical presentation	Biochemical biomarkers	Diagnosis	Treatment	Outcome
Hong et al.2023 (18)	30/M	EC	7d after 2c	Difficulty in opening both eyes and swallowing, minor weakness in their limbs. Then significant limb weakness, dysphagia, hypercapnia, respiratory failure, ptosis, and ocular muscle paralysis.	CK, CK-MB, and cTnI increased.	ICI-related inflammatory myopathy	MPS 2mg/kg/d×5d intravenously,4mg/kg/d×2w orally, then three plasmapheresis + IVIG 400mg/kg/d×5d	Transferred from the NICU to the general ward and discharged.
Zheng et al.2023 (5)	52/M	LCNEC	4m after 5c	Shortness of breath, loss of appetite and weight	CK, CK-MB, and cTnT increased	ICIAM	DXM 15 mg/d, then replaced to MPS 120 mg.	cTnT returned to normal levels 6 months later.
Wang et al.2023 (6)	45/F	Thymoma	14d after 1c	Abdominal pain, chest tightness, dizziness, fatigue and droopy eyelids	cTnI, LDH, CK and CK-MB increased	ICIAM, MG	MPS 120 mg/d×2d, IVIG 25 g/d + MPS 40 mg/d × 3d	Died 11 days after admitted.
Liu et al.2023 (7)	45/F	GC	4w after 1c	Chest pain and palpitations	HSTNI, NTproBNP and CK-MB increased	ICIAM	MPS 120 mg; after impairment of consciousness, seizures, and ventricular fibrillation, given MPS 120 mg + IVIG 20 g.	Discharged after 11 days of hospitalization.
Lin et al.2023 (8)	51/M	LUSC	19d after 1c	Dyspnea, chest tightness, bilateral ptosis, severe fatigue, and generalized myalgia without edema.	Myocardial enzymes were markedly elevated.	ICI-associated myasthenia gravis, myositis, and myocarditis overlap syndrome	MPS 500 mg/d, IVIG 5 g/d, and pyridostigmine.	Discharged after 18 days of hospitalization.
Hu et al.2023 (9)	60/M	LUSC	2c	Without obvious heart-related symptoms.	TnT increased	ICIAM	MPS 1 mg/kg/d × 3d.	He received a right lower thoracoscopic pulmonary lobectomy and 20 days later his TnT and NT-proBNP were significantly elevated.
Yin et al.2022 (10)	71/F	eCCA	1w after 1c	Fatigue and low back pain, limited movement, weakness, and soreness in the bilateral lower extremities, both eyelid ptosis, red transparent urine, and mild dyspnea.	CK, CK-MB, and cTnI, MYO increased	ICI-associated myocarditis and myositis/myasthenia gravis overlap syndrome	MPS 500 mg/d × 5d, and IVIG 0.4 g/kg/d × 5d.	Improved.
Liu et al.2022 (11)	42/M	Thymoma (type B2)	13d after 1c	Fever, chest tightness and suffocation, rapid heart rate.	CK, CK-MB, and hs-CTNI increased	ICIAM, immune hepatitis	MPS 1000 mg × 3d, IVIG 30 g.	Died 4 days after admitted
Liu et al.2022 (12)	66/M	LUAD	3w after 1c	Chest pain, shortness of breath	TnI, CK, CK-MB and NT-proBNP increased	ICIAM	MPS 2mg/kg and IVIG 0.4g/kg/d × 7d.	Improved
Ji et al.2022 (13)	78/M	HCC	21d after 1c	Patient has not complained of any discomfort.	CK and CKMB increased	ICIAM	MPS 2 mg/kg·d × 3d, a temporary cardiac pacemaker was implanted.	Improved
Yang et al.2021 (14)	33/M	Thymoma	1m after 1c	Dyspnea, palpitation, and muscle weakness	TnT, NT-proBNP, and CK increased	ICIAM	MPS 2 mg/kg/d, IVIG 20 g/d × 5d, and pyridostigmine 180 mg/d.	Improved
Liang et al.2021 (15)	77/M	Chordoma	3w after 1c	Acute chest tightness, shortness of breath, sweating profusely, and droopy eyelids on both sides.	BNP, CK-MB, TnT and MYO increased	ICIAM, MG	MPS 160mg q8h × 5d, 80mg q8h × 3d, 40mg q8h × 7d, 40mg q12h × 7d, 40mg qd × 9d, prednisone 50mg.	Improved
Bi et al.2021 (16)	68/M	NSCLC	6d after 3c	Cough and progressive dysphagia	Myocardial enzyme spectrum disturbances	ICIAM	MPS was gradually reduced from 80 to 40 mg/d	Discharged on day 13 after admission.
Xin et al.2020 (17)	66/M	LUAD	4d after 2c	Fatigue, myalgia and tender muscles in both the upper and lower extremities	CPK, MYO, and TnT increased	Immune induced-myositis/myocarditis and rhabdomyolysis.	MPS 2 mg/kg/d, IVIG 400 mg/kg/d × 5d, temporary pace-maker, NIPPV, then MPS 500 mg/d × 5 d and pyridostigmine bromide 120 mg, tracheotomy.	3 months later, he removed from ICU to general ward.

PT, pathological types; M, male; F, female; EC, esophageal cancer; NICU, neuro-intensive care unit; LCNEC, large cell neuroendocrine carcinoma; GC, gastric cancer; LUSC,lung squamous cell carcinoma; eCCA, extrahepatic cholangiocarcinoma; LUAD, lung adenocarcinoma; HCC, hepatocellular carcinoma1;NSCLC, non-small cell lung cancer; m, month; c, cycle; d, day; w, week; ICI, immune checkpoint inhibitors; ICIAM, ICI-associated myocarditis;MG,myasthenia gravis; DXM, dexamethasone; MPS, methylprednisolone; IVIG, immunoglobulin; CK, creatine kinase; CK-MB, creatine kinase isoenzyme; cTnT, cardiac troponin T; NT-ProBNP, terminal-pro B type natriuretic peptide; LDH, lactate dehydrogenase; HSTNI, sensitive troponin I; MYO, myoglobin; hs-CTNI,high-sensitivity troponin; CPK, creatine phosphate kinase; NIPPV, non-invasive positive pressure ventilation.

The clinical manifestations of ICI-induced myocarditis may include fatigue, muscle weakness, myalgias, chest pain, diplopia, ptosis, shortness of breath, orthopnoea, lower extremity oedema, palpitations, lightheadedness/dizziness, syncope, and cardiogenic shock (1). However, the patient in this case report did not exhibit these typical signs, similar to some case reports (9, 13). cTnI is a specific and sensitive biomarker for myocardial injury. A positive troponin test, with troponin I or T levels exceeding the 99th percentile of the upper reference limit, may indicate a cardiovascular irAE (1). In our case, the patient’s cTnI levels were significantly elevated. Given the patient’s lack of previous heart issues but a history of ICI therapy, we strongly suspected ICI-induced myocarditis after ruling out acute myocardial infarction, pulmonary embolism and aortic dissection. Although nab-paclitaxel was used in combination with sintilimab, myocarditis caused by nab-paclitaxel is rare (22, 23).

Myocarditis can present with variable and complex clinical symptoms, making it a diagnostic challenge (24). Patients who have started ICI therapy within the past 8 weeks, have experienced one or more non-cardiac irAEs, and exhibit troponin elevations with atypical trends should be suspected of having myocarditis (24). The diagnosis is suspected based on new cardiac symptoms, new ECG changes, and/or a new increase in troponin levels. Cardiovascular imaging should be performed to confirm cardiac dysfunction and exclude other possible causes (24, 25). Despite being the gold standard for diagnosing ICI-associated myocarditis, endomyocardial biopsy is rarely performed due to the intrusive nature of the procedure and the heightened risk of heart perforation. CMR is considered the premier non-invasive imaging method for myocarditis diagnosis. However, in cases with ICI myocarditis, its sensitivity might be diminished (1). Some scholars also believe that novel biomarkers, including inflammatory and myocardial injury biomarkers, need to be considered in the early diagnosis of myocarditis (21). Regrettably, our patient opted against undergoing the CMR as he didn’t experience any discomfort.

According to the ASCO Guideline (26), our patient falls into the G2 category of Cardiovascular Toxicities. The guideline specifies that in cases of G2 or higher toxicity, immunotherapy with ICIs should be held, and treatment should be discontinued (26). Therefore, the patient discontinued sintilimab treatment. For patients presenting with grade≥ 2 cardiovascular toxicities, the early initiation of high-dose corticosteroids (1-2 mg/kg/d of prednisone) is advisable, as it is likely to be beneficial without AEs (26). Initially, we administered methylprednisolone 40 mg/day for 2 days to the patient, but there was still increase an cTnI levels. Subsequently, the dosage of methylprednisolone was adjusted to 120 mg/day for 4 days. This led to a short-term decline in cTnI levels, which subsequently rose again, accompanied by changes in the patient’s electrocardiogram. According to guidelines, for myocarditis, an intravenous dose of 1000 mg/day of methylprednisolone should be prescribed (1). For patients unresponsive to hormone treatment, the recommendation of other immunomodulatory drugs is advised (1). Consequently, we introduced high-dose steroids with methylprednisolone pulse dosing 1000 mg/day intravenous for 3 days, after which the dose was gradually decreased. The patient’s cTnI levels gradually decreased. However, this was accompanied by the development of several new conditions, including side effects caused by methylprednisolone such as walking weakness, hyperglycemia, oral candidiasis, pneumonia, and even septic shock. In the review of these case reports (5–18), there was rare mention of the side effects associated with methylprednisolone or other immunomodulatory drugs.

For patients needing long-term steroids, especially the elderly, diabetes or immunocompromised, it’s vital to prevent opportunistic infections and implement proactive strategies to manage toxicity (26). Prophylaxis against Pneumocystis jirovecii pneumonia (PJP) should be considered for patients undergoing treatment with a prednisone equivalent of ≥ 20 mg/d for four or more weeks or ≥ 30 mg for three weeks or more. We administered SMZ to the patient on the first day of initiating 120 mg/d methylprednisolone to prevent PJP. However, the patient still experienced pulmonary infections and septic shock. Septic shock, a potentially life-threatening condition, occurs when sepsis leads to cellular metabolism abnormalities and abnormalities low blood pressure. The patient exhibited symptoms including fever, elevated heart rate, tachypnea, leukocytosis, elevated plasma CRP and PCT, arterial hypotension, arterial hypoxemia, and hyperlactatemia, leading to a diagnosis of septic shock (27). The mortality rate of severe sepsis and septic shock is currently approximated at 20 to 30%. Inappropriate or delayed antibiotic treatment correlates with increased mortality (27). Fortunately, timely intervention, including antibiotics, led to patient improvement. However, post-discharge, the patient experienced recurrent fever, pneumonia, and oral candidiasis. Managing steroid-related complications requires a long-term, comprehensive approach. Decision-making should involve a multidisciplinary strategy and consider institutional guidelines.

We present a case of a male patient with recurrent esophageal cancer, who, after receiving sintilimab combination with chemotherapy, subsequently developed immune-related myocarditis. While methylprednisolone treatment ameliorated the myocarditis, it concurrently induced a series of infections. To our knowledge, this report is notable for being the first to document asymptomatic immune myocarditis during the sintilimab treatment of esophageal cancer. It is also the first report to address the side effects of methylprednisolone during the treatment of sintilimab-related myocarditis. However, our report is not without limitations. Primarily, we did not employ CMR to detect myocardial abnormalities. Secondly, initially, the dosage of methylprednisolone administered was that prescribed for cardiovascular toxicities rather than specifically for myocarditis, resulting in a suboptimal initial dose.

## 4 Conclusion

In summary, myocarditis caused by sintilimab is not completely controllable. We should be cautious about ICI-induced myocarditis. For an early diagnosis, routine monitoring is required during treatment. If confirmed myocarditis occurs, intravenous methylprednisolone should be prescribed aggressively and sufficiently. We should also focus on the prevention and management of adverse effects related to steroid use. Meanwhile, more clinical instances must be encountered to gain additional experience.

## Data Availability

The original contributions presented in the study are included in the article/supplementary material. Further inquiries can be directed to the corresponding author.
